# Austin District Medical Society

**Published:** 1891-11

**Authors:** 


### AUSTIN DISTRICT MEDICAL SOCIETY.

By reference to notice elsewhere published in this issue it will be seen that this flourishing society will hold its seventeenth quarterly and fourth annual meeting in this city on Thursday, 17th of December, prox. The program published is very attractive, but in addition to what is there stated, no doubt there will be much of interest in the way of volunteer papers and verbal reports of cases.

We are informed by the indefatigable Secretary, Dr. Bennett, that a large delegation from the Central District Association, which meets at Waco, will be present by invitation; and that several, perhaps four, of the new professors from the Texas Medical College are coming up to take part in the proceedings. Arrangements have been made to take down, stenographically, the discussion; and that on syphilis—the subject set especially for debate—is expected to be very full and interesting; the members are reading up, the Doctor says, in order to take a part. It is hoped to be able to give both the paper (by Dr. Weller) which is to introduce the subject, and the discussion in our next issue.

This association has grown in strength, numbers and interest, steadily, form the first; and it now numbers over one hundred of the most progressive physicians in central and southern Texas. All physicians, accessible to Austin, are cordially invited to be present. The session will wind up with a banquet, as usual, for the doctors are beginning to realize that they need bodily pabulum as well as mental.

